# Surface Enhanced CdSe/ZnS QD/SiNP Electrochemical Immunosensor for the Detection of *Mycobacterium Tuberculosis* by Combination of CFP10-ESAT6 for Better Diagnostic Specificity

**DOI:** 10.3390/ma13010149

**Published:** 2019-12-31

**Authors:** Noremylia Mohd Bakhori, Nor Azah Yusof, Jaafar Abdullah, Helmi Wasoh, Siti Khadijah Ab Rahman, Siti Fatimah Abd Rahman

**Affiliations:** 1 Institute of Advanced Technology, Universiti Putra Malaysia, UPM Serdang 43400, Selangor, Malaysia; noremyliamb@gmail.com; 2 Department of Chemistry, Faculty of Science, Universiti Putra Malaysia, UPM Serdang 43400, Selangor, Malaysia; jafar@upm.edu.my (J.A.); sitikhadijah.fyuz@gmail.com (S.K.A.R.); 3 Faculty of Biotechnology and Biomolecule Science, Universiti Putra Malaysia, Serdang 43400, Selangor, Malaysia; helmi_wmi@upm.edu.my

**Keywords:** tuberculosis, electrochemical immunosensor, plasmonic ELISA

## Abstract

In this study, an electrochemical immunosensor was introduced for the detection of tuberculosis (TB) via utilization of a modified electrode containing a quantum dot (CdSe/ZnS QD) and functionalized silica nanoparticles (SiNPs) on screen-printed carbon electrode (SPCE) CdSe/ZnS QD/SiNPs/SPCE, by employing indirect enzyme-linked immunosorbent assay (ELISA). Here, the fabricated electrode was linked to the biocatalytic action of enzyme catalase through antigen–antibody binding for the detection of the antigen (CFP10–ESAT6) by means of producing a differential pulse voltammetry (DPV) current. The characterization and cyclic voltammetry (CV) of the modified electrode showed good electrochemical behavior and enhanced high electron transfer between the electrode and analyte. Moreover, the active surface area was 4.14-fold higher than the bare SPCE. The developed method showed high selectivity towards CFP10–ESAT6 compared with the other TB proteins. The detection of CFP10–ESAT6 also showed a linear response towards different concentrations of CFP10–ESAT6 with R^2^ = 0.9937, yielding a limit of detection (LOD) of as low as 1.5 × 10^−10^ g/mL for a linear range of 40 to 100 ng/mL of CFP10–ESAT6 concentration. The proposed method showed good reproducibility of target analyte with a relative standard deviation of 1.45%.

## 1. Introduction

Tuberculosis (TB) is the leading cause of death among bacterial infectious diseases [1]. In 2016, approximately 10.4 million people worldwide were diagnosed with TB, of which 1.7 million cases were accounted for TB-related deaths, such as those with tuberculosis-human immunodefiency viruses (TB–HIV) infections. In Malaysia, TB has emerged as a major public health issue [2] according to the statistics released by the Ministry of Health, with cases increasing by 6% from 24,220 in 2015 to 25,739 in 2016 and the annual death toll increasing by 15% from 1696 to 1945, respectively. The high migration rate of foreign workers from TB-stricken neighboring countries, such as Indonesia, Cambodia, Philippines, and Vietnam, were reported to be the main contributors to the spread of TB in this country since it has surpassed the local death rate due to dengue. 

TB is normally caused by airborne bacterial infection from *Mycobacterium tuberculosis* (Mtb) [3]. This infection normally targets the lungs (pulmonary TB), but it could also attack the kidney, brain, and spine. In recent years, several tests have been available for the diagnosis of Mtb, including microscopy, serological test, nucleic acid amplification test (NAAT), and interferon-gamma release assay combined with tuberculin skin test [4,5,6,7]. However, most of these techniques have low sensitivity and specificity, with high-burden settings, which are time-consuming, inconsistent, and involve multiple specimens per patient. Currently, the extensive spread of Mtb, especially in the pulmonary, drug-resistant, and HIV-infected TB cases, lacks accurate tests. 

Hence, there is a need for an efficient system capable of detecting TB-related biomarkers that can provide a quick diagnosis for the immediate treatment of the disease [8]. The detection of TB involves protein detection for target biomarkers, such as the secreted protein antigen 85 complex B (Ag85B), 6-kDa early secreted antigenic target (ESAT6), culture-filtered protein (CFP10), proline-proline-glutamic acid (PPE68), and Mtb protein 64 (MPT64) [9,10,11,12]. Among these antigens, CFP10 and ESAT6 were demonstrated to possess strong antigenicity for T cells that elicit powerful immune responses and protection against Mtb. They were encoded in the genomic region of difference 1 (RD-1), which is present in all the virulent members of the Mtb complex but is absent in BCG vaccine strains. Thus, prior BCG vaccination would not give false-positive results, which is closely related to the virulence of Mtb [13]. Furthermore, Mtb releases a heterodimeric protein complex containing ESAT6 and CFP10 that are essential for the virulence, with the ESAT6 component possessing multiple virulence-related activities [14]. CFP10, which is also known as esxB or Mtb-specific antigen 10 (MTSA 10), contains a C-terminal sequence that enables the secretion of the complex from the bacterial cytoplasm. The complex, in turn, is believed to dissociate under acidic conditions [15]. Among the Mtb antigens, the CFP10 protein has the strongest interaction with cognate antibody, and an early secretory antigen was also found to be abundant in the culture filtrate of Mtb [16]. CFP10 as a TB biomarker could also easily distinguish the Mtb complex from non-tuberculous mycobacteria (NTM) with high sensitivity and specificity in order to maintain good signal intensity for a week [17].

The utilization of antigen-antibody complex in immunonanosensors can improve the drawbacks of the current strategies and increases the performance of diagnostic tools for TB. Generally, immunonanosensors are compact analytical devices that can detect the formation of antigen–antibody complexes and convert them, by means of a transducer, into an electrical signal, which the output can be processed, recorded, and displayed [18]. The types of transducers are classified according to the signal generation such as electrochemical transducer [19,20], optical transducer [21,22], and piezoelectric transducer [23,24]. Electrochemical immunosensors employ the antibody as the capture agent and quantitatively measures the electrical signal resulting from the binding event between the antibody and the target molecule or antigen. The electrical signal often comes from the catalytic reaction of enzyme molecules labelled as a signal tracer with detection antibody [25]. Products containing electric charges can be detected by electrodes, thereby enabling a sensor device measurement for point-of-care (POC) testing [26]. 

This study introduces an electrochemical immunosensor that utilizes a screen-printed carbon electrode (SPCE), which offers various advantages, such as low cost, portability, and simple operation. Nanomaterials, such as silica and quantum dot, can improve the performance of sensing devices due to their unique chemical, physical, and electronic properties. The fabrication of silica nanoparticles with SPCE (SiNPs/SPCE) and CdSe/ZnS quantum dot Si/SPCE (CdSe/ZnS QD/SiNPs/SPCE)-modified electrode is presented as a new strategy to improve the electrochemical immunosensor for the detection of CFP10–ESAT6 protein using the differential pulse voltammetry (DPV) technique. It is expected that utilizing SiNPs with CdSe/ZnS QD on the electrode surface can improve the function of the sensing device for good electrocatalytic performance. To date, the utilization of a combination of SiNPs and CdSe/ZnS QD as a modifier in the electrochemical sensor for CFP10–ESAT6 detection has not been reported. The electrochemical sensing method based on SiNPs and CdSe/ZnS QD was employed in this study owing to their high sensitivity and selectivity, low cost, portability, and short analytical time measurement of CFP10–ESAT6 for POC requirement in TB diagnosis.

## 2. Materials and Methods

### 2.1. Materials

A 1× phosphate-buffered saline (PBS; 0.1 mM, pH 7.4) solution was prepared by dissolving a 1× PBS tablet in 100 mL deionized water (obtained with a Thermo Scientific water system, Barnstead Nanopure, Waltham, MA, USA) for dilution of all antigens with antibodies. *Mycobacterium tuberculosis* ESAT-6-like protein esxB (CFP10–ESAT6)) with antibodies (mouse monoclonal anti-CFP10–ESAT6 and goat polyclonal secondary antibody to mouse IgG2b (Biotin)) were purchased from Abcam Sdn. Bhd. (Kuala Lumpur, Malaysia). A wash buffer solution was freshly prepared with PBS and tween-20. A blocking solution was freshly prepared with PBSA containing PBS and 1% Bovine serum albumin (BSA). PBS, tween-20, BSA, ammonium hydroxide, 30% (NH_4_OH), tetraethyl orthosilicate (TEOS), lumidot CdSe/ZnS quantum dot (CdSe/ZnS QD), toluene, thioglycolic acid (TGA), 3-aminopropyltriethoxysilane, and 98% (APTES) were purchased from Sigma-Aldrich (St. Louis, MO, USA). Ethanol, 95% (EtOH), was the product of HmBG Chemicals (Hamburg, Germany). PBS in tablet form was obtained from Amresco (Radnor, PA, USA).

### 2.2. Instruments

Electrochemical measurements were performed using a µAUTOLAB (III) electrochemical potentiostat (Eco Chemie, Utrecht, The Netherlands) using screen-printed junction cable controlled by General Purpose Electrochemical System software version 4.9 and NOVA 1.11 software. Both methods were operated using SPCE consisting of three electrode systems, a working electrode, a carbon counter electrode, and an AgCl reference electrode immersed in a glass medium containing the supporting electrolyte and analyte. The reference electrode was made of silver. The diameter of the working electrode was 4 mm and the geometric working area was 0.11 cm^2^. The electrodes were all printed on a ceramic substrate (3.4 cm long × 1.0 cm wide × 0.05 cm thick). Fluorescence spectroscopy was performed using Shimadzu RF-5301PC (Kyoto, Japan). Field emission scanning electron microscopy (FESEM) was carried out using JSM 7600F, JEOL (Tokyo, Japan) microscope equipped with an energy-dispersive X-ray (EDX) system (Hitachi S-3400N, Tokyo, Japan) while high-resolution transmission electron microscopy (HRTEM) was from JEM-2100F transmission electron microscope (Santa Clara, CA, USA). Fourier transform-infrared spectroscopy (FTIR) studies were executed using 100 series PerkinElmer (Waltham, MA, USA).

### 2.3. Preparation of CdSe/ZnS QD/SiNPs/SPCE

Firstly, 4 mL of EtOH and 3.3 mL of NH_4_OH were mixed under stirring. Then, 4 mL of TEOS was added to the mixture and left for 24 h. Next, 0.3 mL of APTES was added to the mixture and left overnight. The mixture was centrifuged at 40,000 rpm for 2 h and then dried in an oven at 70 °C for 30 min. The prepared SiNPs were ground using pastel and mortar. For further usage, 3 mg of SiNPs was mixed with 1 mL of deionized water and sonicated for 1 h.

Water-soluble CdSe/ZnS QD was prepared by modification of its hydrophobic surface with TGA. Briefly, the mixture of 1 mL CdSe/ZnS QD in toluene and 1 mL EtOH was centrifuged at 3000 rpm for 15 min. Then, the precipitate was washed with EtOH. These steps were repeated twice, after which the precipitate was collected and re-dissolved in toluene. Next, 150 µL TGA was added into the solution and sonicated for 30 min, and the mixture was kept at room temperature for 24 h. Afterwards, the solution was centrifuged at 5000 rpm for 5 min, unwanted solution was discarded, and the precipitate was dissolved in 1× PBS three times before being dried in a desiccator for 1 h and finally re-dissolved in 1× PBS.

SiNPs/SPCE was prepared by dropping 15 µL of SiNPs solution on the working electrode of SPCE and then incubating for 1 h and 30 min at room temperature. Meanwhile, for CdSe/ZnS QD/SiNPs/SPCE, CdSe/ZnS QD was first activated with EDC and NHS and incubated for 1 h. Next, 1 µL of the activated CdSe/ZnS QD was dropped on the SiNPs/SPCE and then dried for overnight. 

### 2.4. Immunoassay Procedures

The immunoassay procedures for CdSe/ZnS QD/SiNPs/SPCE were constructed by dropcasting 5 µL of 0.12 µg/mL of antigen (CFP10–ESAT6) onto the modified electrode and then incubating overnight at 4 °C. Then, the unreacted antigen was washed away by dipping in PBS solution twice before being dried on a hot plate at 37 °C. Next, the electrode was blocked with 1% BSA for 2 h at room temperature followed by washing again with PBS and drying on the hot plate. Subsequently, a 5 µL of 0.5 µg/mL primary antibody was again dropcast on the electrode, incubated for 2 h at room temperature, washed with PBS and dried at 37 °C. Thereafter, a 5 µL of 0.5 µg/mL secondary antibody was dropped on the electrode and incubated at room temperature for 2 h, washed with PBS, and dried again. Lastly, a 5 µL of streptavidin-catalase conjugate was dropped on the electrode and incubated for another 2 h, followed by drying of the electrode. The procedures were followed with an operated DPV used for the detection procedures in 150 µM H_2_O_2_ containing 1× PBS (pH 7.4). The same procedures were studied for negative detection without the addition of CFP10–ESAT6 in the immunoassay procedures. 

Selectivity and sensitivity studies were investigated by separate immobilization of 20 ng/mL of CFP10–ESAT6 and another TB biomarker protein, MPT64, and 1 mg/mL BSA on the CdSe/ZnS QD/SiNPs/SPCE, followed by immunoassay procedures described in the previous section. Furthermore, sensitivity studies were also conducted at different concentrations of the CFP10–ESAT6 (20, 40, 60, 80, and 100 ng/mL). The DPV procedures were operated for both selectivity and sensitivity studies in 150 µM of H_2_O_2_ containing 0.1 mM PBS (pH = 7.4) at –1.0 to 1.0 V potential. The reproducibility study was conducted to evaluate the reproducibility of the entire experiment. By using the same modification method, the reading of five different CdSe/ZnS QD/SiNPs/SPCE modified electrode was taken, followed by the calculation of the average and standard deviations

## 3. Results and Discussion

### 3.1. Functionalized Silica Nanoparticles

Functionalized silica nanoparticles (SiNPs) were obtained from slightly modified of well-known Stober method, in which TEOS and ammonia served as precursor and catalyst, respectively [27]. Firstly, colloidal SiNPs were prepared that involved two types of reactions; (1) formation of silanol groups by hydrolysis and (2) formation of siloxane bridge by condensation polymerization reaction [28]:

Si‒(OR)_4_ + H_2_O ↔ Si‒(OH)_4_ + 4R‒OH, (1)

2Si‒(OH)_4_ → 2(Si‒O‒Si) + 4H_2_O. (2)

**Scheme 1:**
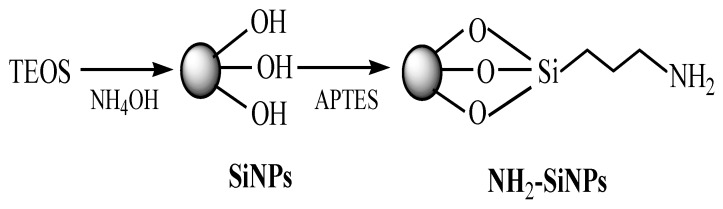
Preparation of amine-functionalized silica nanoparticles (SiNPs).

Secondly, SiNPs were converted into amine-functionalized SiNPs by reaction of colloidal SiNPs with 3-aminopropyltriethoxysilane (APTES) [29]. Silanes are attached through the formation of a Si-O-Si bond between the surface and the silanol groups. The bond formation between SiNPs surface and the APTES molecules proceeds with the hydrolysis of the alkoxyl groups followed by the covalent adsorption of the hydroxysilane product, resulting in the formation of aminopropyl silane [30]. Then, the alkoxy group of APTES binds to the hydroxyl group of SiNPs leading to the presence of free amines (NH_2_). Scheme 1 shows the preparation of amine-functionalized SiNPs.

**Figure 1:**
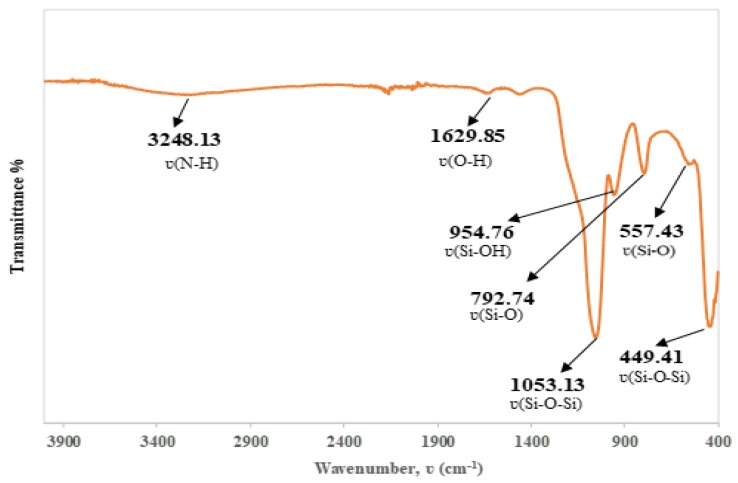
Fourier transform-infrared (FTIR) spectra of functionalized SiNPs.

The FTIR spectrum in Figure 1 of SiNPs shows that the absorption bands at 3248.13 and 1629.85 cm^−1^ were attributed to the stretching mode of NH_2_ and OH, respectively. Furthermore, the characteristic bands at 1053.13, 954.76, and 792.74 cm^−1^ corresponded to the stretching vibration of Si–O–Si, Si–OH, and Si–O, which are known as the characteristic bands of silica. The peaks at 557.43 and 449.41 cm^−1^ indicated the stretching vibration of Si–O and the bending vibration of Si–O–Si [31]. Clearly, all these bands confirmed the successful synthesis of functionalized SiNPs.

### 3.2. Surface Characterization of Modified Electrode

**Figure 2:**
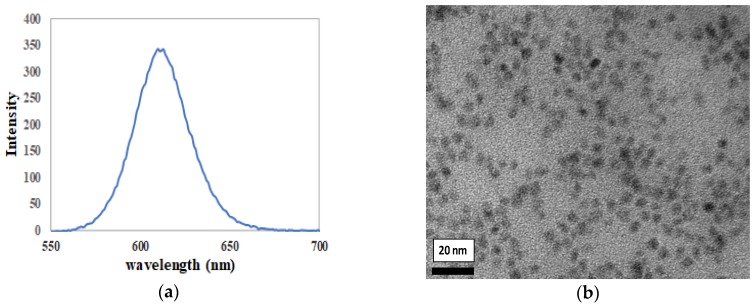
(**a**) Fluorescence emission of CdSe/ZnS QD excited at 365 nm; (**b**) high-resolution transmission electron microscopy (HRTEM) of CdSe/ZnS QD at magnification of 200 k.

The commercialized semiconductor CdSe/ZnS QD contained oleic acid and trioctylphosphine (TOPO) as organic ligands. Introduction of COOH groups enhanced the compatibility of QD to covalently bind with NH_2_ groups on protein and SiNPs. The hydrophobic ligands around surface of CdSe-ZnS QD were replaced by hydrophilic ligand from thioglycolic acid (TGA), which contains thiol (-S) functional groups that attached at the surface of CdSe-ZnS QD. The strong affinity of the thiolated groups to the CdSe-ZnS QD will cause the electrostatic binding of the hydrophilic ligands to the surface of CdSe-ZnS QD. Moreover, modification of CdSe/ZnS QD with TGA enhanced the fluorescence intensity because of the simple-chain or non-bulky groups of the TGA molecules [32]. Figure 2a shows the fluorescence emission of CdSe/ZnS QD at 615 nm with excitation of 365 nm. HRTEM of CdSe/ZnS QD in Figure 2b shows the semiconductor of nanoparticles to be uniformly dispersed with each other as single particles without any aggregation even after modification with TGA.

**Figure 3:**
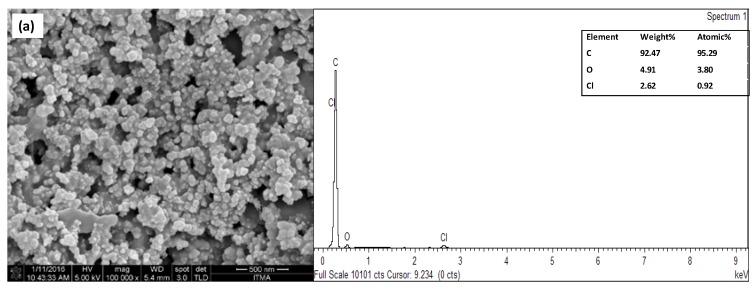

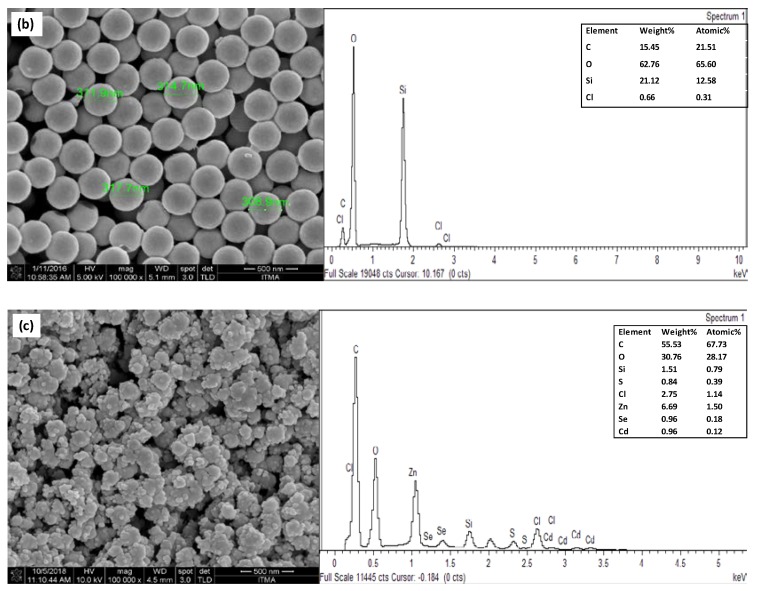
Images of field emission scanning electron microscopy (FESEM) under 100 k magnification and EDX of (**a**) bare screen-printed carbon electrode (SPCE), (**b**) SiNPs/SPCE, and (**c**) CdSe/ZnS QD/SiNPs/SPCE.

Figure 3a shows the FESEM image for the distribution of carbon particles on the surface of a bare SPCE, whereas the SiNPs/SPCE-modified electrode clearly showed the SiNPs to be well distributed and covering the surface of SPCE (Figure 3b). Figure 3c shows the image of the CdSe/ZnS QD/SiNPs/SPCE-modified electrode. However, it can be observed that SiNPs were not well distributed due to the agglomeration of the nanomaterial on the surface of SPCE. CdSe/ZnS QD was not clearly seen on the image of CdSe/ZnS QD/SiNPs/SPCE because FESEM was not a suitable analysis to observe the image of CdSe/ZnS QD on the electrode; nonetheless, the presence of CdSe/ZnS QD elements on the surface of electrode can be assured by EDX analysis of the desired electrodes. Based on the spectral image, there was one sharp peak on the bare SPCE due to the carbon relative to the electrode surface (Figure 3a). Moreover, the appearance of silicon (Si) and oxygen (O) peaks at the spectral image in Figure 3b confirmed that the SiNPs were widely dispersed on the SPCE and that the carbon (C) peak was due to the carbon electrode from which the sample was dropcast onto. The spectral image in Figure 3c shows that the peak of cadmium (Cd), selenium (Se), zinc (Zn), and sulphur (S) belonged to CdSe/ZnS QD, which assured the deposition of CdSe/ZnS QD on the SPCE electrode, although FESEM did not show the image of CdSe/ZnS QD on the CdSe/ZnS QD/SiNPs/SPCE-modified electrode.

### 3.3. Electrochemical Characterization of SiNPs/SPCE and CdSe/ZnS QD/SiNPs/SPCE

**Figure 4:**
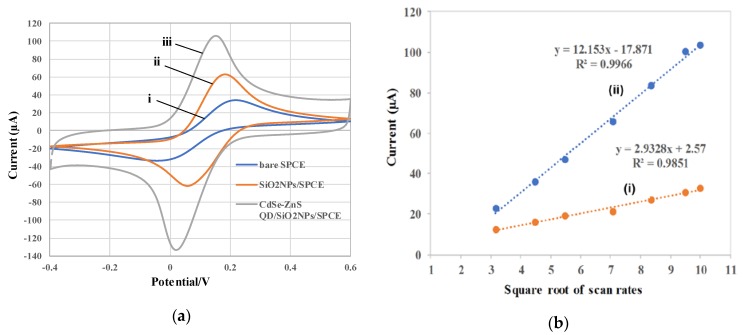
(**a**) Cyclic voltammetry (CV) responses for (i) bare SPCE, (ii) modified SiNPs/SPCE, and (iii) modified CdSe/ZnS QD/SiNPs/SPCE in 5.0 mM K_3_Fe(CN)_6_ solution containing a supporting electrolyte, 0.1 M KCl, at 100 mV/s; (**b**) plot of the oxidation peak current of (i) bare SPCE and (ii) CdSe/ZnS QD/SiNPs/SPCE in 5.0 mM K_3_Fe(CN)_6_ solution containing supporting electrolyte 0.1 M KCl at different scan rates (mV/s).

The cyclic voltammetry (CV) technique was employed to study the electrochemical behavior of fabricated electrodes in potassium ferricyanide, K_3_Fe(CN)_6_ solution, which is a good redox probe to provide the electron transfer behavior since the Fe(CN)_6_^3−/4−^ ion can provide fast electrochemical responses [33]. CV responses for the electrodes are shown in Figure 4a in 5.0 mM K_3_Fe(CN)_6_ containing 0.1 M KCl solution as a supporting electrolyte at a scan rate of 100 mV/s with the potential window of 0.6 to −0.4 V. Accordingly, bare SPCE (Figure 4a (i)) showed the lowest redox peak current, compared with SiNPs/SPCE and CdSe/ZnS QD/SiNPs/SPCE. The redox peak current and electrode performance of SPCE were greatly increased after modification with SiNPs, as shown in Figure 4a (ii), because of the large surface area of SiNPs that increased the active surface area of the modified electrodes. Furthermore, the CdSe/ZnS QD/SiNPs on the electrode surface highly enhanced the peak current responses due to the fast electron transfer provided by CdSe/ZnS QD, as shown in Figure 4a (iii). In addition, a small peak-to-peak separation for CdSe-ZnS QD/SiNPs/SPCE (+0.16 V) was less compared to bare (+0.23 V) and SiNPs/SPCE (+0.19 V), which indicated a faster kinetics of electron transfer rate, which results from the strong interaction between SiNPs and CdSe-ZnS QD, which enhance the electron transfer between analyte and electrode surface [34]. 

Meanwhile, the active surface area can be calculated according to the Randles–Sevcik equation as I_pa_ = (2.687 × 10^5^) n^3/2^ V^1/2^ D^1/2^ AC; where I_pa_ (A) represents the anodic peak current, n is the number of electron transfers, V (Vs^-1^) is the scan rate, D (cm^2^·s^−1^) is the diffusion coefficient (7.6 × 10^-6^ cm^2^·s^−1^) [35], A is the electroactive surface area, and C (mol/cm^3^) is the concentration of K_3_Fe(CN)_6_. The estimation of effective surface area for electrode is important in monitoring the performance of modified electrode for electrochemical sensor development. A larger surface area can increase the efficiency of electroactive site that can be exposed for electrocatalytic reaction. The experiments were performed using CV techniques at different scan rates (10–100 mV/s). Figure 4b shows that cathodic peak currents were directly proportional to the square root of the scan rate (v½), indicating the diffusion-controlled redox process [36]. The active surface area of bare SPCE and CdSe/ZnS QD/SiNPs/SPCE was calculated at 0.0792 and 0.3281 cm^2^, respectively, which means that the active surface area of CdSe/ZnS QD/SiNPs/SPCE was 4.14-fold higher than bare SPCE. The CdSe/ZnS QD/SiNPs/SPCE-modified electrode showed an increment of active surface area compared with bare SPCE and indicated that the fabrication of the modified electrode clearly enhanced the effective surface area of the developed sensor.

### 3.4. Immunoreaction for CFP10–ESAT6 Detection using DPV

Catalase (H_2_O_2_:H_2_O_2_ oxidoreductase, EC 1.11.1.16) from different sources exhibit similar molecular weight and number of subunits including bovine liver catalase, which is the enzyme in tetramer with a total molecular weight of approximately 240,000 [37]. Each tetramer molecule is composed of four heme groups in which the iron ion is in the ferric state [38,39]. Heme-containing catalases break down H_2_O_2_ by a two-step mechanism. First, catalase heme Fe^3+^ reduces one molecule of H_2_O_2_ to water and generates a covalent Fe^4+^ =O called oxyferryl species and a porphyrin cation radical, both referred to as compound I [40]. At the second step, compound I oxidizes a second H_2_O_2_ molecule to regenerate the enzyme and forming oxygen and water molecules [41]. The catalytic mechanism of catalase is shown below:

Heme Fe^3+^ + H_2_O_2_ → E (^α+^ por)-Fe^4+^ [Compound I] + H_2_O, (3)

[Compound I] + H_2_O_2_ → Heme Fe^3+^ + O_2_ + H_2_O. (4)

**Figure 5:**
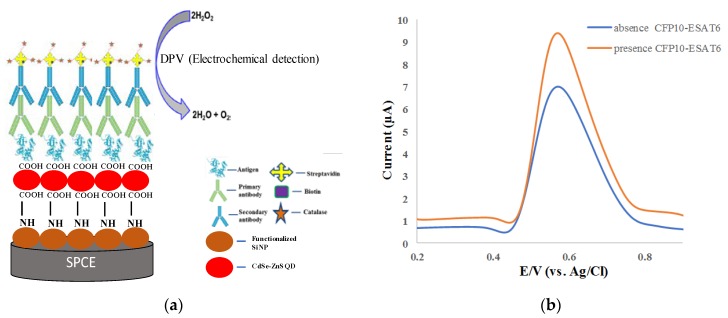
(**a**) Schematic diagram of electrochemical immunoreaction for differential pulse voltammetry (DPV) response of oxidation of H_2_O_2_ to produce water molecule and oxygen catalyzed by catalase for the detection of CFP10–ESAT6; (**b**) DPV current response of H_2_O_2_ oxidation for immunoassay in the absence and presence of 0.12 µg/mL CFP10–ESAT6 protein in 0.1 M PBS (pH = 7.4) containing 150 µM H_2_O_2_.

An electrochemical immunosensor was developed for the detection of CFP10-ESAT protein as TB biomarker utilized indirect ELISA assay. In this format, SPCE was modified with functionalized SiNPs that contain amine (NH_2_) functional groups (SiNPs/SPCE) for attachment with carboxyl groups (COOH) of CdSe/ZnS QD. The modified electrode (CdSe/ZnS QD/SiNPs/SPCE) was dropcast with the target protein as antigen and then captured by primary antibody. The secondary antibody conjugated with enzyme catalase via biotin–streptavidin linkage binds with the primary antibody. The fabricated electrode was measured by DPV in PBS solution containing hydrogen peroxide (H_2_O_2_) as electrolyte. The presence of catalase of the CdSe/ZnS QD/SiNPs/SPCE-modified electrode resulted in high-oxidation-peak current of DPV response as the catalase catalyzed the decomposition of H_2_O_2_ to produce water molecules and oxygen. However, in the absence of antigen, there was no catalase present on the electrode since there was no binding between the primary antibody and secondary antibody–catalase conjugate. Thus, decomposition of H_2_O_2_ to produce water molecule at a slow rate resulted in lower DPV oxidation peak current. Figure 5a shows the schematic diagram of electrochemical immunoreaction for DPV response of the H_2_O_2_ oxidation to produce water molecules and oxygen catalyzed by catalase for the detection of antigen. 

Figure 5b shows the DPV of modified electrodes. An oxidation peak occurred at +0.56 V for both peaks, which indicates the reduction current peak of H_2_O_2_. In the absence of CFP10–ESAT6 protein, there was no catalase in the system, which contributed to a decrease in the peak current. Afterwards, the complete immobilization of indirect ELISA system in the presence of CFP10–ESAT6 protein resulted in an increase in oxidation peak current contributed by the biocatalytic reduction of H_2_O_2_ by catalase to produce water molecule and O_2_. Additionally, the electron flow was facilitated by high electroconductivity and has increased the surface area of CdSe/ZnS QD/SiNPs/SPCE [42].

### 3.5. Selectivity, Sensitivity, and Reproducibility Study

**Figure 6:**
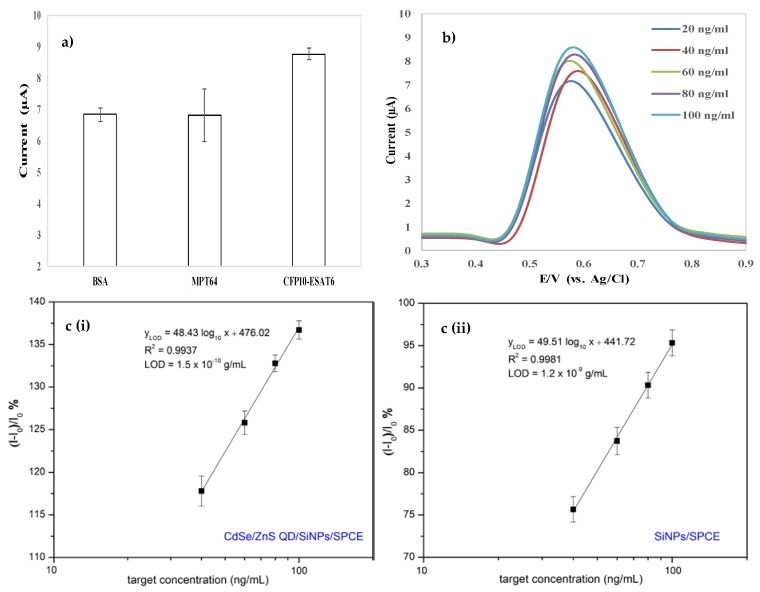
(**a**) Oxidation peak current of H_2_O_2_ by using different types of protein (1% BSA, 20 ng/ml MPT64, and 20 ng/mL CFP10–ESAT6) in 0.1 M PBS (pH 7.4) containing 150 µM H_2_O_2_; (**b**) DPV current response of oxidation H_2_O_2_ versus different concentrations of CFP10–ESAT6; (**c**) calibration curve of the immunosensor towards different CFP10–ESAT6 concentrations in 0.1 M PBS (pH 7.4) containing 150 µM H_2_O_2_ using (**i**) CdSe/ZnS QD/SiNPs/SPCE and (**ii**) SiNPs/SPCE.

Figure 6a shows the high DPV current for CFP10–ESAT6 protein. Meanwhile, the DPV response reduced to almost half for both BSA and MPT64 proteins and obviously produced about the same DPV current value. The lower current for both proteins was the result of the protein of BSA and MPT64 not binding with the primary antibody and thus caused the absence of the catalase at the top of the ELISA system for catalytic reaction with H_2_O_2_. However, the DPV current response for BSA and MPT64 were generated due to the modified electrode of CdSe/ZnS QD/SiNPs/SPCE increasing the effective surface area of the electrode and resulting in the peak current. MPT64 was chosen as another biomarker for the specificity study because it is a protein secreted by actively growing Mtb strains and is only found in viable and actively dividing cells of Mtb. Moreover, the utilization of MPT64 was suitable because it is one of the secreted Mtb antigens besides CFP10 (10 kDa) and ESAT6 (6 kDa) with low molecular weight (28 kDa). The value of DPV current for MPT64 as one of the mycobacterial antigens located in RD2 of Mtb genome is about the same as that of BSA, a known type of common protein used in ELISA for blocking and as diluent. This result indicated that the newly developed electrochemical biosensor by the authors can discriminate proteins other than the CFP10–ESAT6 protein. Figure 6b demonstrates the DPV current response of oxidation H_2_O_2_ by the different concentrations of CFP10–ESAT6 in the range of 20–100 ng/mL.

The correlation between the sensor responses and the target concentrations as obtained in Figure 6c indicates that the detection responses of the electrode were linear with the value of the target CFP10–ESAT6 in the concentrations ranged of 40–100 ng/mL. The measurement of the test was repeated three times for every concentration of the CFP10-ESAT6. The detection response of the developed sensor can be expressed as the relative change in current (ΔI/I_0_), where ΔI is the change in peak current after the target detection and I_0_ is baseline current before detection [43]. The limit of detection (LOD) was calculated using the 3 sigma method; Y_(LOD)_ = 3σ, where σ is the residual standard deviation [44]. The associated concentration for LOD of the CdSe/ZnS QD/SiNPs /SPCE was approximately 1.5 × 10^−10^ g/mL, indicates that low concentrations of target protein can effectively be detectable. Remarkably, the detection limit demonstrated was clearly improved compared to the SiNPs/SPCE with detection limit of 1.2 × 10^−9^ g/mL, proving that the sensor performance could be enhanced by modified the electrode using CdSe/ZnS QD/SiNPs. Since the physiological range of interest of CFP10-ESAT6 is around 100 ng/mL in TB patients [45], the low detection limit of this sensor platform was applicable for the practical application in TB diagnosis. This approach compared reasonably in terms of linearity range and LOD with other previously reported TB biomarkers detections, as summarized in Table 1.

Note that in the reported literature, CFP10 and ESAT6 were used as antigens for TB detection. In the developed immunosensor herein, the combination of CFP10 and ESAT6 (CFP10–ESAT6) exhibited the ability to improve the diagnostic specificity without loss of sensitivity and thus offers a realistic alternative for the diagnosis of TB in clinical purpose [52]. In addition, in combination with CFP10, ESAT6 is able to enhance the permeability of artificial membranes by disrupting the lipid bilayers and acts as cytolysin, while exposed C-terminal region of CFP10 may be involved in interaction with a host cell target protein resulting in stabilization of the helical conformation [53]. This implies that, without its partner CFP10, ESAT6 might not be active [54] and the virulence of Mtb is reduced by the knockout of either ESAT6 or CFP10; thus, the heterodiamer is important in the virulence of Mtb. Renshaw and group also stated that the complex CFP10–ESAT6 acts as a signal molecule that lead to the complex being a target in diagnosis of TB [15]. 

In addition, reproducibility in the immunosensor was very important for confirming the reliability of the developed method. A series of five electrodes was prepared for the detection of 100 ng/mL CFP10–ESAT6 protein. Table 2 presents the results, which demonstrated good reproducibility of the CdSe/ZnS QD/SiNPs/SPCE. The mean, standard deviation (SD), and relative standard deviation (RSD) of the measurements for the five electrodes were 8.264%, 0.1203%, and 1.45%, respectively.

## 4. Conclusions

We have demonstrated a practical approach to develop a simple electrochemical-based SPCE for TB detection, employing CFP10-ESAT6 as protein biomarker. An interesting platform based on a combination of CdSe/ZnS QD and SiNPs was presented in order to amplify the detection signal as well as to increase the selectivity of the sensor towards the TB-specific biomarkers. The EDX spectral showed the existence of Cd, Se, Zn, and S elements on the modified surface electrode, which confirmed the deposition of CdSe/ZnS QD on the surface. The active surface area of CdSe/ZnS QD/SiNPs/SPCE was calculated to be 4.14-fold higher than bare SPCE. Moreover, the responsivity of the developed sensor was thoroughly investigated by observing the peak current change in response to the presence of CFP10-ESAT6. It was observed that a linear calibration curve was constructed in the range of 40–100 ng/mL of target concentration, with the limit of detection for CdSe/ZnS QD/SiNPs/SPCE reduced to 1.5 × 10^−10^ g/mL as compared to 1.2 × 10^−9^ g/mL for SiNPs/SPCE. The procedure described herein can provide a good reproducibility with the RSD value of 1.45%, proving that this analytical assay holds great potential to be used as point-of-care disease diagnostics tool.

## Figures and Tables

**Table 1:** The comparison of analytical performance of developed electrochemical immunosensor by various protein tuberculosis (TB) biomarkers.

Method	Linear Range	LOD	Tuberculosis Biomarker	References
DPV	0.00002–1 ng/mL	1.0 × 10^−7^ g/mL	MPT64	[46]
DPV	5–500,000 ng/mL	3.3 × 10^−10^ g/mL	CFP10	[47]
EIS	0.0001–0.1 ng/mL	1.2 × 10^−4^ g/mL	IFN-ɣ	[48]
SWASV	0.001–0.5 ng/mL	3.4 × 10^−4^ g/mL	IFN-ɣ	[49]
EIS	0.0001–0.02 ng/mL	3.3 × 10^−5^ g/mL	ESAT6	[50]
DPV	20–100 ng/mL	1.5 × 10^−8^ g/mL	CFP10	[51]
SWV	10–1000 ng/mL	7.0 × 10^−9^ g/mL	ESAT6	[10]
DPV	20–100 ng/mL	1.5 × 10^−10^ g/mL	CFP10-ESAT6	This work

DPV: Differential pulse voltammetry, EIS: Electrochemical impedance spectroscopy, SWASV: Square wave anodic stripping voltammetry, SWV: Square wave voltammetry, LOD: Limit of detection.

**Table 2:** Reproducibility of 100 ng/mL of CFP10–ESAT6 protein.

Replicate	Peak Current (ng/mL)
1	8.14
2	8.22
3	8.36
4	8.42
5	8.18
**Mean**	8.264
**SD**	0.1203
**RSD**	1.4561
